# A Survey of Aflatoxin-Producing *Aspergillus* sp. from Peanut Field Soils in Four Agroecological Zones of China

**DOI:** 10.3390/toxins9010040

**Published:** 2017-01-20

**Authors:** Chushu Zhang, Jonathan Nimal Selvaraj, Qingli Yang, Yang Liu

**Affiliations:** 1 Institute of Food Science and Technology, Chinese Academy of Agricultural Sciences/Key Laboratory of Agro-Products Processing, Ministry of Agriculture, Beijing 100193, China; zcs.2003@163.com (C.Z.); sjonnim@gmail.com (J.N.S.); 2 Shandong Peanut Research Institute, Qingdao 266100, China; 3 Qingdao Agricultural University, Qingdao 266109, China; rice407@163.com

**Keywords:** aflatoxin B_1_, aflatoxin-producing *Aspergillus*, peanut soils, China

## Abstract

Peanut pods are easily infected by aflatoxin-producing *Aspergillus* sp.ecies from field soil. To assess the aflatoxin-producing *Aspergillus* sp. in different peanut field soils, 344 aflatoxin-producing *Aspergillus* strains were isolated from 600 soil samples of four agroecological zones in China (the Southeast coastal zone (SEC), the Yangtze River zone (YZR), the Yellow River zone (YR) and the Northeast zone (NE)). Nearly 94.2% (324/344) of strains were *A. flavus* and 5.8% (20/344) of strains were *A. parasiticus*. YZR had the highest population density of *Aspergillus* sp. and positive rate of aflatoxin production in isolated strains (1039.3 cfu·g^−1^, 80.7%), the second was SEC (191.5 cfu·g^−1^, 48.7%), the third was YR (26.5 cfu·g^−1^, 22.7%), and the last was NE (2.4 cfu·g^−1^, 6.6%). The highest risk of AFB_1_ contamination on peanut was in YZR which had the largest number of AFB_1_ producing isolates in 1g soil, followed by SEC and YR, and the lowest was NE. The potential risk of AFB_1_ contamination in peanuts can increase with increasing population density and a positive rate of aflatoxin-producing *Aspergillus* sp. in field soils, suggesting that reducing aflatoxigenic *Aspergillus* sp. in field soils could prevent AFB_1_ contamination in peanuts.

## 1. Introduction

Peanuts *(Arachishypogaea L)* are an important economic crop in China, with its annual production being the highest on a global level at 16 million tons in 2015. China also accounts for more than 40% of world peanut production [1,2]. The four agroecological zones, namely the Southeast coastal zone (SEC), Yangtze River zone (YZR), the Yellow River zone (YR) and the Northeast zone (NE), are the major peanut producing regions in China [3].

Peanuts are often infected during pre-harvest by *Aspergillus* sp. which produces aflatoxins that are carcinogenic to humans and animals [4,5]. Soil is the main source of inoculum for aflatoxigenic *Aspergillus* sp.ecies, and since peanut pods grow underground, they are in direct contact with the soil fungal population [5,6].

Chemically, aflatoxins belong to the bisfuranocoumarin group, with aflatoxins B_1_ (AFB_1_), B_2_, G_1_, G_2_ being the most common contaminants. Aflatoxins have the potential to cause outbreaks of acute hepatitis and even liver cancer in animals and humans [7,8]. Of the naturally occurring aflatoxins, AFB1 is known for its toxic and carcinogenic nature [9,10]. In China, AFB_1_ predominantly contaminates peanuts, with an average rate of 86.2% of the total aflatoxins and a correlation coefficient of 0.99 [11].

Though aflatoxin-producing *Aspergillus* infection occurs generally on the aerial section of the host plant, soil tends to be the key reservoir for its inoculum and aflatoxin contamination in peanuts. This means it is definitely essential to assess the level of aflatoxin-producing *Aspergillus* sp. in field soils, which are prone to infecting peanuts when grown in these regions. Differences in the aflatoxin-producing *Aspergillus* communities in the agroecological zones are of great importance to understand their population dynamics and helping to develop suitable control measures for aflatoxin contamination reduction in the fields [12,13] .

In China, risk assessments of dietary exposure to AFB_1_ in post-harvest peanuts have been conducted [12,14], but little information exists on the distribution of aflatoxin-producing *Aspergillus* in the soil from different agroecological zones where peanuts are cultivated. Furthermore, no studies have been conducted on the AFB_1_ producing potential of *Aspergillus* in field soils in these regions.

The aim of this work was to examine the distribution and AFB_1_ producing ability of aflatoxin-producing *Aspergillus* across the four agroecological zones in peanut-producing soils in China and to determine the reason behind contamination.

## 2. Results

### 2.1. Distribution of Aflatoxin-Producing Aspergillus across China

Aflatoxin-producing *Aspergillus* sp.ecies were isolated from all the 600 soil samples collected from the four agroecological zones in China. In total, 344 *Aspergillus* strains were isolated from all the soil samples (Table 1). Among them, 324 strains (94.2%) were identified as *A. flavus* and the remaining 20 strains (5.8%) were identified as *A. parasiticus*. The population of aflatoxigenic *A. flavus* was highly predominant among the fungal population collected from all the districts.

A significant difference in the population density and positive rate of aflatoxin-producing *Aspergillus* sp. across the agroecological zones was observed (Table 2). YZR zone (1039.3 cfu·g^−1^, 80.7%) was the highest, followed by SEC (191.5 cfu·g^−1^, 48.7%) and YR (26.54 cfu·g^−1^, 22.7%). But the population density and positive rate of aflatoxigenic *Aspergillus* sp. in the NE zone (2.4 cfu·g^−1^, 6.6%) was significantly less than the other three reported zones in this study. The population densities of aflatoxigenic *Aspergillus* sp. varied among the districts from all the zones, ranging from 1.1 to 2749.3 cfu·g^−1^. In SEC zone, the Yunfu district recorded the highest population densities of aflatoxin-producing *Aspergillus* with high positive rate (481.1 cfu·g^−1^, 66.7%), but was significantly less in Shantou district (19.9 cfu·g^−1^, the positive rate 36.7%). When compared to SEC zone, population densities and positive rate of aflatoxigenic *Aspergillus* sp. in YZR zone was significantly higher, with Xiangzhou district (2749.3 cfu·g^−1^, 90%) being the highest and the lowest was Xiaogan district (55.5 cfu·g^−1^, 33.3%). But in YR zone, they were significantly lower when compared to SEC and YZR zone, with the Qingdao district (52.2 cfu·g^−1^, 30.0%) being the highest and Linyi (5.7 cfu·g^−1^, 16.7%) as the lowest. NE zone recorded significantly lower population densities of aflatoxin-producing *Aspergillus* and positive rate among all the zones studied, the largest district was Tieling (4.4 cfu·g^−1^, 13.2%), and the least districts were Dalian (1.1 cfu·g^−1^, 3.31%) and Shenyang (1.1 cfu·g^−1^, 3.31%). The population density had a significant positive correlation with the positive rate (*r* = 0.65). Also, the positive rate had a significant positive correlation with temperature (*r* = 0.61), but was negatively correlated with longitude (*r* = −0.71), and pH had a great influence on the positive rate (*r* = 0.48) (Table 3).

**Figure 1:**
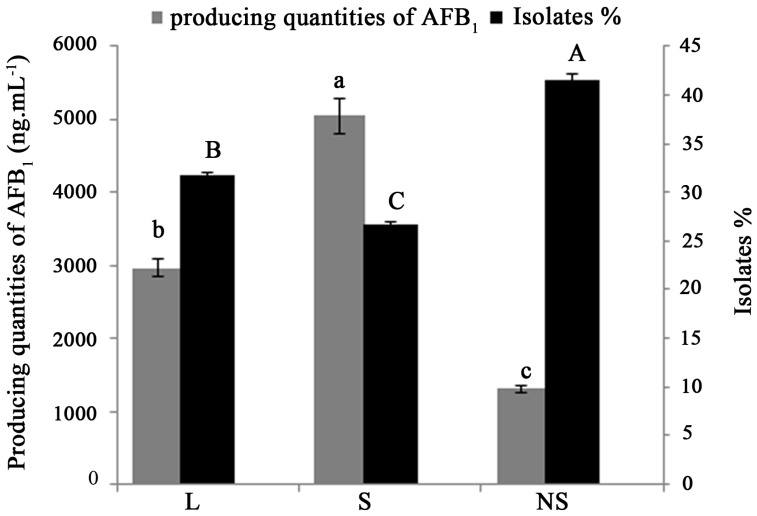
Distribution and producing quantities of AFB_1_ of different phenotype of aflatoxin-producing *Aspergillus* in peanut fields of China. For each bar, vertical lines represent the standard error of the mean; means not sharing a common letter are significantly different according to Tukey’s HSD test (*p* = 0.05).

**Figure 2:**
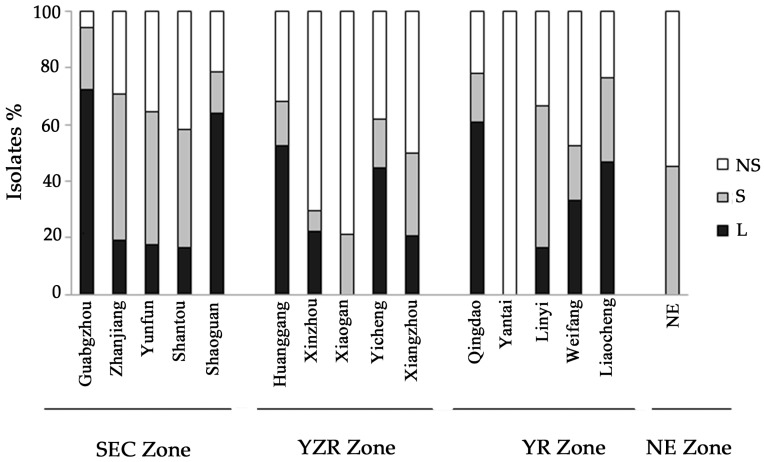
Distribution of different phenotype of aflatoxin-producing *Aspergillus* sp. among districts.

The isolates were further grouped according to sclerotium production and size: L-strain (average diameter >400 μm), S-strain (average diameter <400 μm) and NS (non-sclerotial)-strain (Figure 1). The NS-strains were recorded in highest percentage, representing nearly half of all the isolated strains from all the soil samples. In contrast, the recoveries of L-and S-strains were only 31.7 and 26.7%, respectively. Statistical analysis showed a significant difference in percentage of L, S and NS-strains (*p* ˂ 0.05). Figure 2 shows the distribution of different aflatoxin-producing *Aspergillus* morphotype among the sampling districts. Frequencies in the percentage of L, S and NS-strains significantly varied within the districts in each zones. L-strains were not observed in Xiaogan district from YZR zone, Yantai district from YR zone, and NE zone, S -strains were not observed in Yantai district from YR zone, but NS strains were observed in all the zones. NS strains were found to be significantly lower in Guangzhou district from SEC zone than in other districts. S strains were found to be significantly lower in Xiaogan district from YZR zone than in other districts. However, not much of a significant difference was found among the L strains between the zones. The average production of AFB_1_ by different *Aspergillus* phenotypes (Figure 1) was significantly different, with the highest being S-strains (5047.5 ng·mL^−1^), followed by L-strains (2963.4 ng·mL^−1^), and NS-strains (1304 ng·mL^−1^).

### 2.2. Distribution of Aflatoxin-Producing Aspergillus Chemotypes

**Figure 3:**
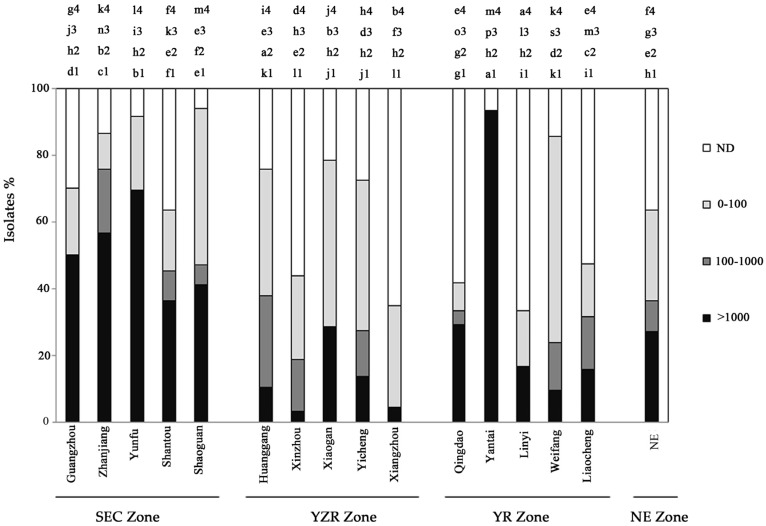
Variation of among districts in the percent of aflatoxin-producing *Aspergillus* isolates producing quantities of AFB_1_. ND, none AFB_1_ detected; 0–100, producing of AFB_1_ by isolates was 0–100 ng·mL^−1^; 100–1000, producing of AFB_1_ by isolates was 100–1000 ng·mL^−1^; >1000, production of AFB_1_ by isolates was >1000 ng·mL^−1^; lines not sharing a common letter are significantly different (*p ≤* 0.05) according to Tukey’s HSD test on ranks of AFB_1_ concentrations.

**Figure 4:**
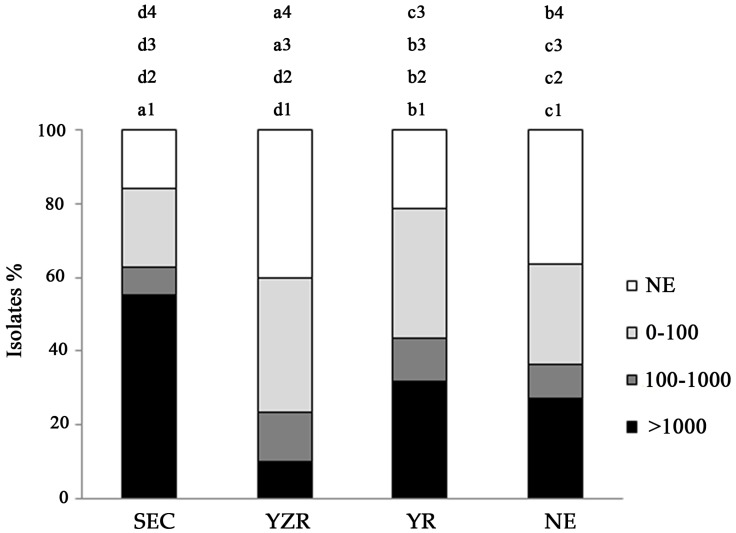
Variation of among four agroecological zones in the percent of aflatoxin-producing *Aspergillus* isolates producing quantities of AFB_1_ in China. ND, none AFB_1_ detected; 0–100, production of AFB_1_ by isolates was 0–100 ng·mL^−1^; 100–1000, producing of AFB_1_ by isolates was 100–1000 ng·mL^−1^; >1000, producing of AFB_1_ by isolates was >1000 ng·mL^−1^; a line not sharing a common letter is significantly different (*p ≤* 0.05) according to Turkey’s HSD test on ranks of AFB_1_ concentrations.

Frequencies of AFB_1_ production varied among the sampling districts (Figure 3) and agroecological zones (Figure 4). The Yantai district from YR zone had a significantly larger proportion (93.3%) of aflatoxigenic strains (>1000 ng·mL^−1^) with higher percentage of *A. parasiticus* (Table 1), compared to all the districts in all zones. The second largest population of highly aflatoxigenic isolates was seen in Yunfu district (69.4%) in SEC zone. Xinzhou and Xiangzhou districts in YZR zone recorded the lowest aflatoxigenic populations with 4.3% and 3.1%, respectively. Among the four agroecological zones, the highest proportion of isolates producing >1000 ng·mL^−1^ level of aflatoxin was observed in SEC zone (55.4%) which was 5.4 times higher than other zones. The YZR zone had the lowest percentage of isolates (10.2%) producing >1000 ng·mL^−1^ of aflatoxin.

Aflatoxin-producing potential varied among isolates, species, districts, and agroecological zones (Table 2). All the tested *A. parasiticus* isolates produced AFB_1_. The average production of AFB_1_ by the *A. flavus* isolates was 3420 ng·mL^−1^ (range: 0–82,083 ng·mL^−1^), which was significantly lower than AFB_1_ production of *A. parasiticus* isolates with an average of 14,780 ng·mL^−1^ (range: 5.8–61,899 ng·mL^−1^). *A. flavus* contributed the most to the average aflatoxin-producing potential in fungal community resident in twelve districts (Table 2). *A. parasiticus* made a greater contribution to the aflatoxin-producing potential of fungal communities in the districts of Zhanjiang, Qingdao, and Yantai, and in NE zone. According to Pearson’s correlation analysis (Table 3), there was a significant positive correlation between the average aflatoxin-producing potential of fungal communities and percentage of the isolates which produced high levels of AFB_1_ (>1000 ng·mL^−1^) (*r* = 0.87, *p* = 0.01).

The average AFB_1_ producing potential varied widely among the zones. Average AFB_1_ producing potential of *Aspergillus* isolates in the SCE zone (5795 ng·mL^−1^) showed higher significance than the YZR zone (1566 ng·mL^−1^), but not much of a significant difference from the YR zone (5453 ng·mL^−1^). The average AFB_1_-producing potential of *Aspergillus* isolates in the NE zone (1886 ng·mL^−1^) was the least significant compared to other zones. Within the zones, the highest average of AFB_1_ concentration was observed in Yantai district (18,784 ng·mL^−1^) from YR zone, and the least in Xinzhou district (73 ng·mL^−1^) from YZR zone (Table 2).

The potential risk areas with respect to aflatoxigenic *Aspergillus* sp. and their AFB_1_ production varied among four agroecological zones (Table 4). Based on the AFB_1_ production, its highest level was seen in SEC zone, where the average production from the isolates taken from 1 gram of soil was 5795 ng·mL^−1^ (Table 4). The second was YR zone, with an average AFB_1_ production of 5453 ng·mL^−1^. The least were NE and YZR zones, where the average aflatoxin production level was lower in isolated strains. As soil is the main reservoir of aflatoxin-producing *Aspergillus* inoculums and aflatoxin contamination in peanuts, the survey of *Aspergillus* sp. and their aflatoxin production from 1 gram of soil could reflect the potential risk of contamination. The potential peanut production can be at a higher risk of AFB_1_ contamination in YZR zone based on the survey, as no isolates in 1 gram of soil were reported as the highest, and least in the NE zone.

## 3. Discussion

This study provided the first comprehensive documentation of the potential risk of aflatoxin contamination on peanuts by aflatoxigenic *Aspergillus* sp. in soils from the major peanut producing regions of China. Although aflatoxigenic *Aspergillus* strains have been reported from various crops and agricultural commodities, agricultural soil serves as the main reservoir of these fungi all over the world [15,16,17,18]. In the present study, *A. flavus* was the dominant species of *Aspergillus* fungal population in peanut soil of all districts, which was 94.2% of all strains. Aflatoxin-producing *Aspergillus* sp. was seen in all districts. In Brazil, *A. flavus* was the most frequent species of the genus *Aspergillus* in soil samples from four peanut production regions (13.4%) [19]. In Argentina, the *Aspergillus* population recovered from peanut seeds showed *A. flavus* as the most frequently isolated (79%) strain [20]. Not only in peanuts, *A. flavus* is the predominant species in the soils and vegetables of corn, cotton and other crops [7,21,22,23,24]. Also *A. flavus* was widely seen reported in temperate and tropical regions [25,26].

The aflatoxin-producing *Aspergillus* present in all fields, as estimates from dilution plating showed that the population ranged from 1.1 to 2749.315 cfu·g^−1^ (315 cfu·g^−1^), which was higher than the population reported from soil in Argentina’s peanut-growing region(212 cfu·g^−1^) [4], but lower than the *Aspergillus* population from soil in Nigeria’s maize-growing region (1150 cfu·g^−1^) [27] and soil of Lowa’s corn-growing region (1231 cfu·g^−1^) [28].

*A. parasiticus* was isolated in all four agroecological regions. *A. parasiticus* is usually found reported in sugar cane, grapes or cassava from tropical and subtropical regions. In SEC zone, which is a tropical and subtropical region, *A. parasiticus* strains had been isolated from Zhanjiang (Sugar cane as rotating crop) and Yunfu districts (Cassava as rotating crop), which was consistent with the previous studies. But *A. parasiticus* isolated in the field soils of Yicheng district from YZR zone, Qingdao, Yantai, and Weifang districts from YR zone and Fuxin, and Shenyang from NE zone had maize as the rotating crop. We speculate that *A. parasiticus* may have had a transmission route with corn as the medium, and the transmission extended from tropical and subtropical to the warm temperate zone and temperate zones.

Sclerotia have been demonstrated to be the survival structure for many fungi. Because sclerotia of aflatoxin-producing *Aspergillus* can germinate sporogenically, they could be a potential source of primary inoculums. In this study, nearly 60% of the isolates produced sclerotia, which suggest that it is essential for the survival of aflatoxigenic *Aspergillus* in the peanut ecosystem in China, together with mycelia and conidia. These results are similar to previous studies [4,29].

From the soil samples of China’s peanut-growing region, the NS-strains were isolated in the highest percentage and the S-strains with the lowest percentage. Barros [4] reported the isolation of L-strains in highest percentage from soil samples of peanut-growing region in Argentina. Soil isolates of *A. flavus* along a transect within the United States were identified as members of either the L-strains (*n* = 774), or the S-strains (*n* = 309) [13]. Orum [30] reported ranges of S strain incidence from less than 5% to more than 90%, and the association with cotton cultivation in the southern United States. In Arizona, incidence of S strains is inversely correlated with elevation [31].

So far, researchers have done some research on the effect of crop rotation on *A. flavus* types. Nesci and Etcheverry [32] recovered only *A. flavus* L phenotype from insects, soil and debris samples from corn fields alternately cropped with peanuts and soybeans from the same region. In our study, the isolation frequency of the L-strains within the aflatoxin-producing *Aspergillus* was higher. The L phenotype was recovered in the highest percentage in the districts of Guangzhou, Shaoguan, Huanggang, Yicheng, Qingdao and Liaocheng, and the crop rotation in these six districts were either rice and corn (rice, rice, corn, corn, corn, and corn respectively), so the rotation between corn or rice towards peanut was conducive to the growth and reproduction of the L phenotype. On the other hand, Horn et al. [33] and Cotty et al. 12 found that the S strain was widely distributed in cotton-producing areas in the United States. The condition responsible for the S strains distribution appeared to be complex. In the fields along a transect through the peanut-growing region of the USA described by Horn and Donner 13, the S strain was highly prevalent in west central Texas and Louisiana, where cotton is grown extensively. These studies and our results suggest that the crop could be selecting the phenotype found, as was reported by Garber [3] and Barros [4].

The soil type, landform and rainfall had a greater influence on the growth of aflatoxin-producing *Aspergillus* in different agroecological zones. In SEC zone, the top three districts with a higher population density of aflatoxigenic *Aspergillus* sp. and positive rate were Yunfu (481.1 cfu·g^−1^, 66.7%), Shaoguan (258.6 cfu·g^−1^, 46.7%) and Zhanjiang (170 cfu·g^−1^, 56.7%), and the soil types of these districts were all arid hillside, and the least was observed in Guangzhou (27.8cfu·g^−1^, the positive rate 36.7%) and Shantou (19.9 cfu·g^−1^, the positive rate 36.7%) where the soil type was paddy soil. The rainfall in the southeast coastal region is more due to the subtropical monsoon climate, and paddy soil has poor drainage, resulting in soil with high water content which is not conducive to the growth of aflatoxigenic *Aspergillus.* However, the hillside had good drainage and water retention, which produced suitable soil moisture for the growth of *A. flavus*. In YZR zone, the district with the highest population density and positive rate was Xiangzhou (2749.3 cfu·g^−1^, 90%), the second was Huanggang district (1920 cfu·g^−1^,100%), and the least was observed in Xiaogan (55.5 cfu·g^−1^, 33.3%). High temperatures and a drought period with very little rainfall were observed before two months of peanut harvest in the YZR zone. Meanwhile, the agrotype of Xiangzhou was clay loam and the landform of Huanggang district was plains, which were beneficial to maintaining soil moisture for the growth of aflatoxin-producing *Aspergillus.* In Xiaogan, the agrotype was sandyloam and the landform was arid hillside, which all were detrimental to water retention, so soil moisture in Xiaogan was too low to inhibit the growth of aflatoxin-producing *Aspergillus* sp.

The majority of aflatoxin-producing *Aspergillus* isolates from peanut soil across the four agro-ecological zones was aflatoxigenic. In previous studies, the average aflatoxin-producing potential of fungal communities highly varied. In the southern USA [13,34] and in Argentina’s peanut the majority of *Aspergillus* isolates produced aflatoxins [35]. While in Iran, only 27.5% of aflatoxin-producing *Aspergillus* isolates from corn soil were toxigenic [36], and in Nigeria [27], 44% of aflatoxin-producing *Aspergillus* isolates were aflatoxigenic. Different results of aflatoxigencity among Aspergillus section Flavi population may be attributed to differences in prevailing climatic conditions, the cultivar grown, and local agricultural practices.

We observed a positive association between aflatoxin production and sclerotia phenotype in *A. flavus* isolates from China peanut soil since the S strains produce higher levels of aflatoxin than the L strain isolates and similar results were found in Argentina [4,37]. These results are supported by previous studies that showed an evident interrelationship between regulation, biosynthesis and sclerotia morphogenesis [4,38].

Orum et al. [30] postulated that temperature, soil condition, day length, crop sequence history, insect levels, rainfall frequency and management practice may influence aflatoxin-producing *Aspergillus* communities. All these factors and many other micro-climatic factors are different between these four agroecological zones of China. In the present study, the population density had a significant positive correlation with positive rate (*r* = 0.65). Positive rate of *Aspergillus* sp. had a significant positive correlation with temperature (*r* = 0.61), and a significant negative correlation with longitude (*r* = −0.71), had positive correlation with pH (*r* = 0.48). The incidences of the L-strain had significant positive correlation with soil pH and had a negative correlation with latitude. In Nigeria, the incidences of the L-strain also had a significant negative correlation with latitude [27].

The AFB_1_ producing potential isolates in field soils significantly varied among the four peanut production areas. YZR had the largest number of AFB_1_ producing potential isolates, while the least was NE. Ding [39] researched the distribution of AFB_1_ contamination in post-harvest peanut in China, and the highest was observed in the Yangtze River ecological region and the lowest in NE. In our present study, the Yangtze River ecological region had the largest AFB_1_-producing potential isolates in 1 g soil, and AFB_1_ contamination in post-harvest peanut in this region was also reported to be higher. Meanwhile, NE had the least AFB_1_ producing potential isolates in 1 g soil, and AFB_1_ contamination in post-harvest peanut in this region was also lowest. AFB_1_ contamination in post-harvest peanuts was closely related to the AFB_1_ producing potential of peanut fields. Therefore, we drew the conclusion that AFB_1_ contamination risk mainly came from aflatoxin-producing *Aspergillus* sp. in peanut field soils. We can predict that the highest risk zone for AFB_1_ contamination in peanuts is YZR zone, followed by SEC and YR zone. However, NE zone tends to be highly safe for peanut cultivation.

The potential risk of AFB_1_ contamination in peanuts will increase with an increase in population density and positive rate of aflatoxin-producing *Aspergillus* strains in peanut field soils. YZR had the higher population density and positive rate of aflatoxin-producing *Aspergillus* strains (1039.3 cfu·g^−1^, 80.7%), the next highest were SEC (191.5 cfu·g^−1^, 48.7%) and YR (26.5 cfu·g^−1^, 22.7%), and the last was NE (2.4 cfu·g^−1^, 6.6%). These results suggest that the reduction in the number of aflatoxin-producing *Aspergillus* strains in field soil is crucial for controlling AFB_1_ contamination in peanuts. Novel biological control technology has been developed in recent years that can prevent AFB_1_ contamination in peanuts. The application of non-toxigenic *A. flavus* strains to the peanut crop seems to be one of the most efficient strategies [40,41].

## 4. Conclusions

This study has shown that *A. flavus* is the dominant species in peanut soil fungal population in all the agroecological zones, with widespread aflatoxigentic strains. The YZR zone is highly prone to AFB_1_ contamination risk in peanuts, as the study has shown that it has the highest *Aspergillus* sp. population density with a positive rate of aflatoxin production. However, the number of *A. parasiticus* identified was lower compared to *A. flavus*, though, their presence should not be overlooked, as they indicate the possibility of high-risk exposure due to their high level of AFB_1_ production. The influence of AFB_1_ through peanuts on human populations in China over the past decade demonstrates a clear need for tools to manage contamination of locally produced peanuts. Given the widespread nature of AFB_1_- producing strains, any control strategy should include field interventions.

## 5. Materials and Methods

### 5.1. Survey Sites

Soil samples were collected from peanut fields across four agroecological zones: Southeast coastal (SEC), the Yangtze River (YZR), the Yellow River (YR), and the Northeast (NE). Five districts were selected for study sites within each agroecological zones, and these which districts were separated geographically by at least 50 km. Field ecology information for each sampling region are shown in Table 5.

### 5.2. Survey Methods

Six hundred soil samples from fifteen districts (30 from each district) were collected at harvest time. Each soil sample (100 g) consisted of a pool of five subsamples taken with a trowel from the top 5 cm of soil at 5–10 m intervals. The samples were placed in plastic bags with pinholes for gas exchange and transferred to the laboratory and stored at 4–5 °C for further assay [4].

### 5.3. Strain Isolation and Identification

Ten grams of soil from each of the total 600 soil samples were diluted with 90 mL of 0.1% (*w*/*v*) peptone water and kept at room temperature (25 ± 2°C) for 20 min on a thermostatic shaker (Hunan Xiangyi Instrument Co., Ltd, Changsha, China. This mixture was decimally diluted and a 0.1 mL aliquot was spread on dichloran-18% glycerol (DG18) at appropriate dilution to allow collection of isolates from plates with fewer than 10 colonies. The plates were incubated in the dark for 5–7 days at 30 °C. At the end of the incubation period, the average number of duplicate colonies was determined and the results were expressed as colony-forming units per gram (cfu·g^−1^) of soil. Isolates were sub-cultured at 30 °C on malt extract agar (MEA).

All isolates were preliminarily identified based on their characteristic growth patterns on AFPA (*A. flavus* and *A. parasiticus* agar) [43] and CYA (Czapek yeast autolysis) [44]. The identities of the strains were further confirmed by molecular analysis, which involved sequencing of their calmodulin genes [44]. The calmodulin gene in each isolate was amplified using the primers CL1 (GARTWCAAGGAGGCCTTCTC) and CL2A (TTTTTGCATCATGAGTTGGAC).

### 5.4. Classification of Aflatoxin-Producing *Aspergillus* morphotypes

To induce the production of sclerotia, the strains were incubated in Czaper medium, in triplicate, and maintained in darkness at 30 °C for 14 days. Following this period, Tween 20 (100 μL/L, 5 mL) was added, and the surface was scraped so that the mycelia could be filtered through Whatman No.2 filter paper. The sclerotia were washed in distilled water and placed in microtubes until further analysis [37]. To measure the mean diameter of the sclerotia for each strain, 10 sclerotia from each replicate were randomly selected and the arithmetic mean was calculated.

### 5.5. Mycotoxin Analyses

The liquid fermentation method used by Barros [4] was modified and used for qualitative determination of AFB_1_ production by aflatoxin-producing *Aspergillus*. The strains were induced to sporulate on MEA slants at 28 °C for 5 days. After incubation, 5 mL of Sterilized distilled water was added to the slant followed by vigorous agitation to obtain a spore suspension. The spore concentration was measured using a cell counting plate and adjusted to 10^6^ spores mL^−1^. Approximately 10^5^ spores were used to inoculate 50 mL vials containing 10 mL of liquid medium made by dissolving 150 g of sucrose, 20 g of yeast extract, and 10 g of soytone, in 1 L of distilled water; the pH of the medium was adjusted to 6.0 with HCl. One vial was incubated per isolate and performed in triplicate. The cultures were incubated for 7 days at 30 °C with 200 rpm in the dark. Vial cultures were analyzed by high-performance liquid chromatography for the production of AFB_1_. HPLC analysis was performed using Waters 2695 (Waters Corporation, Milford, MA, USA) coupled to Waters 2475 fluorescence detector (λ exc 360 nm; λ em 440 nm) and a post-column derivation system, and an Agilent TC-C18 column (250 × 4.6 mm, 5 μm particle size). The mobile phase (water:methanol:acetonitrile, 4:1:1) was pumped at a flow rate of 0.5 mL/min. AFB_1_ procured from Sigma-Aldrich (St. Louis, MO, USA) was used as standard. The mean recovery of the method used was calculated by culture medium at different levels ranging from 0.5 to 100 ng/g of AFs and was estimated at 95.2% ± 8.4%. The lowest detection limit was 0.5 ng of AFB_1_ per mL [45].

### 5.6. Statistical Analysis

Analyses were performed with SPSS (version 18.0). Analysis of variance was performed on all data with the general linear model (GLM), suitable for unbalanced data. The GLM of SPSS uses the least-squares method to fit data to a general linear model. Tukey’s honestly significant difference (HSD) test was performed to compare treatment means at the 5% level. Pearson’s correlation coefficients were generated to assess relationships between ecological and biological variables.

## Figures and Tables

**Table 1:** Distribution of aflatoxin-producing *Aspergillus* in peanut fields from four agroecological zones of China ^a^.

AZE ^b^	District	%A.f ^c^	%A.p ^d^	Number of Isolates	cfu·g^−1^ Soil		Positive Rate (%) ^e^	Soil pH	Soil Moisture (%)
					Range	Mean			
SEC	Guangzhou	100	0	20	0–399.6	27.8f	36.7h	6.28	21.08
	Zhanjiang	94	6	32	0–1666.7	170e	56.7e	5.13	15.35
	Yunfu	94	6	33	0–5667.7	481.1c	66.7d	5.36	18.05
	Shantou	100	0	13	0–133.3	19.9f	36.7h	6.12	20.15
	shaoguan	100	0	17	0–2333.3	258.6d	46.7f	6.32	14.06
YZR	Huanggang	100	0	29	33.3–6660	1920b	100a	6.86	19.20
	Xinzhou	100	0	32	0–1000	174.3e	83.3c	5.66	17.53
	Xiaogan	100	0	14	0–666.7	55.5f	33.3j	4.95	7.30
	Yicheng	97	3	29	0–1000	297.5d	96.7a	6.79	11.29
	Xiangzhou	100	0	23	0–16,665	2749.3a	90b	6.55	12.57
YR	Qingdao	92	8	24	0–300	52.2f	30.0i	4.78	7.84
	Yantai	47	53	15	0–233.3	16.7f	23.3j	5.30	14.52
	Linyi	100	0	6	0–66.7	5.7f	16.7k	5.21	18.41
	Weifang	90	10	21	0–200	24.4f	40.0gh	5.90	5.90
	Liaocheng	100	0	19	0–166.7	33.7f	43.3fg	7.18	7.18
NE	Dalian	100	0	1	0–33.3	1.1f	3.3l	5.50	13.90
	Jinzhou	100	0	4	0–33.3	2.2f	6.7l	6.38	5.31
	Fuxin	0	100	1	0–66.7	3.3f	6.7l	5.53	5.11
	Shenyang	50	50	2	0–33.3	1.1f	3.3l	5.42	2.50
	Tieling	100	0	3	0–33.3	4.4f	13.2k	4.7	13.24

^a^ Means determined by Tukey’s HSD test (α = 0.05); values in a column followed by a different letter are significantly different; ^b^ SEC, Southeast coastal zone; YZR, the Yangtze River zone; YR, the Yellow River zone; and NE, the Northeast zone; ^c^ %A.f was the percentage of *A. flavus* in all isolates; ^d^ %A.p was the percentage of *A. parasiticus* in all isolates; ^e^ Positive rate (%) was the percentage of the soils which can isolate *A. flavus* in 30 soil sample of each district.

**Table 2:** Quantities of AFB_1_ produced by aflatoxin-producing *Aspergillus* isolated among four agroecological zones of China ^a^.

AZE ^b^	District	Aflatoxin B_1_ (ng·mL^−1^)				
		*A. flavus*		*A. parasiticus*		Average ^c^
		Range	Mean	Range	Mean	
SEC	Guangzhou	0–25,300	8473a	-	-	8473b
	Zhanjiang	0–25,812	5536d	238–20,172	10,324b	5788d
	Yunfu	0–82,083	6493c	70–1380	725c	6173cd
	Shantou	0–16,501	4178f	-	-	4178e
	shaoguan	0–26,293	3092h	-	-	3092f
	**Total**		**5805A**	**-**	**5465C**	**5795A**
YZR	Huanggang	0–17,512	1220j	-	-	1220gh
	Xinzhou	0–1089	73m	-	-	73i
	Xiaogan	0–28,970	7611b	-	-	7611bc
	Yicheng	0–34,870	1803i	5.8	5.8c	1741g
	Xiangzhou	0–2662	147m	-	-	147i
	**Total**		**1579C**	**-**	**5.8D**	**1566D**
YR	Qingdao	0–30,588	3493g	4154–13,923	9039b	3955ef
	Yantai	0–23,225	8343a	7111–61,899	27,920a	18784a
	Linyi	0–2312	392l	-	-	392hi
	Weifang	0–60,331	3734f	14.6–57.5	36c	3381ef
	Liaocheng	0–8640	710k	-	-	710ghi
	**Total**		**3041B**	**-**	**20,125A**	**5453B**
NE	NE	0–3278	366	159–17,293	8726	1886
	**Total**	**0–3278**	**366D**	**159**–**17,293**	**8726B**	**1886C**

^a^ Means with by Turkey’s HSD test (α = 0.05); in a column followed by a different letter are significantly different; ^b^ SEC, Southeast coastal zone; YZR, the Yangtze River zone; YR, the Yellow River zone and NE, the Northeast zone; ^c^ Mean aflatoxin of all isolates.

**Table 3:** Pearson’s correlation coefficients of relationships among the quantity of AFB_1_ producing *Aspergillus* in soil (cfu·g^−*1*^), positive rate %, the proportion of *Aspergillus* isolates with AFB_1_ producing ability ˃1000 ng·mL^−^^1^, 100–1000 ng·mL^−1^, 0–100 ng·mL^−^^1^ , the proportion of L-stain, S-strain and NS-strain, the average AFB_1_ quantification, the potential risk of AFB_1_ contamination (ng·mL^−^^1^/g soil), average temperature (TEM), the longitude (LONG), latitude (LAT), pH and soil moisture (%).

	cfu·g^−1^	%Positive Rate	% ˃ 1000	%100–1000	%0–100	AFB_1_	AFB_1_ Risk	%L	%S	%NS	TEM	%LONG	%LAT	pH	% Moisture
cfu·g^−1^	1.00														
%Positive rate	0.65 **	1.00													
% ˃ 1000	−0.34	−0.26	1.00												
%100–1000	0.13	0.37	−0.40	1.00											
%0–100	0.17	0.10	−0.51	0.21	1.00										
%L	0.07	0.23	−0.20	0.23	0.14	1.00									
%S	−0.06	−0.18	0.04	−0.07	−0.21	−0.31	1.00								
* NS	−0.03	−0.11	0.17	−0.17	0.00	−0.78 **	−0.35	1.00							
AFB_1_	−0.31	−0.32	0.87**	−0.41	−0.36	−0.25	−0.31	0.45	1.00						
AFB_1_ risk	0.39	0.50 *	0.29	0.18	0.08	0.08	0.15	−0.18	0.05	1.00					
TEM	0.33	0.61 *	−0.06	0.15	0.19	0.24	−0.22	−0.09	−0.01	0.42	1.00				
%LONG	−0.06	−0.38	−0.33	−0.01	0.03	−0.26	−0.23	0.40	−0.08	−0.46	−0.64	1.00			
%LAT	−0.36	−0.71 **	0.03	−0.14	−0.21	−0.30	−0.10	0.36	0.18	−0.48	−0.76 **	0.78 **	1.00		
pH	0.42	0.48	−0.41	0.43	0.30	0.49	−0.20	−0.35	−0.39	0.06	0.21	−0.06	−0.28	1.00	
%moisture	0.15	0.35	0.27	−0.02	−0.34	0.11	0.15	−0.20	0.10	0.36	0.42	−0.64 **	−0.29	0.09	1.00

Correlation significance，* 0.01 < *p* < 0.05 ，** *p* < 0.01; *n* = 16.

**Table 4:** Variation among agroecological zones of China for colony-forming units of aflatoxin-producing *Aspergillus*, positive rate, the mean quantities of producing AFB_1_ of isolated strains, potential producing AFB_1_ ability in soil ^a^.

AEZ	No. of Fields	CFU/g Soil	%Positive Rate	%Af ^b^	%Ap ^b^	No. of Tested Isolates	Aflatoxin B_1_^c^(ng·mL^−1^)
SEC	5	191.5b	48.7b	96.6a	3.4c	121	5795a
YZR	5	1039.3a	80.7a	99.1a	0.9d	127	1566c
YR	5	26.5c	22.7c	85.9b	14.1b	85	5453d
NE	5	2.4d	6.6d	81.8b	18.2a	11	1886b

^a^ Means with by Tukey’s HSD test (α = 0.05); values in a column followed by a different letter are significantly different; ^b^ Af% means the proportion of *Aspergillus flavus*; Ap% the proportion of *Aspergillus parasiticus*; ^c^ Mean aflatoxin of all isolates.

**Table 5:** Eco-environmental information of peanut fields.

AEZ ^a^	District	Latitude (N)	Longitude (E)	Temp ^b^	Agrotype	Landform	Alternative Crop
SEC	Guangzhou	23.158029	113.273165	27.81	Sandy loam	Paddy soil	Rice
	Zhanjiang	21.377219	110.25017	27.94	Sandy loam	Arid hillside	Sugarcane
	Yunfu	22.768595	111.570011	28.08	Sandy loam	Arid hillside	Sweet potato
	Shantou	23.285832	116.726481	26.62	Sandy loam	Paddy soil	Rice
	Shaoguan	24.682728	113.604549	26.88	Sandy loam	Paddy soil	Rice
YZR	Huanggang	30.64299	114.872866	28.69	Sandy loam	Plain	Cotton
	Xinzhou	30.841401	114.801259	28.65	Sandy loam	Plain	Cotton
	Xiaogan	31.562299	114.128097	28.40	Sandy loam	Arid hillside	Sweet potato
	Yicheng	31.719806	112.257788	26.74	Sandy loam	Plain	Maize
	Xiangzhou	32.087779	112.211772	27.69	Clay loam	Mound	Maize
YR	Qingdao	35.79045	118.627918	25.02	Sandy loam	Arid hillside	Maize
	Yantai	37.387331	121.60049	25.47	Sandy loam	Arid hillside	Maize
	Linyi	35.8725	120.04643	25.70	Sandy loam	Arid hillside	Sweet potato
	Weifang	36.706945	118.829914	26.44	Sandy loam	Arid hillside	Maize
	Liaocheng	36.866062	116.231478	26.38	Sandy loam	Arid hillside	Maize
NE	Dalian	38.950245	121.565873	22	Sandy loam	Arid hillside	Vegetable
	Jinzhou	41.117250	121.128323	21	Sandy loam	Arid hillside	Maize
	Fuxin	42.065175	121.757901	19.5	Sandy loam	Arid hillside	Maize
	Shenyang	42.74995	123.353519	20	Sandy loam	Arid hillside	Maize
	Tieling	42.785798	124.111098	20	Sandy loam	Arid hillside	Maize

^a^ SEC, Southeast coastal zone; YZR, the Yangtze River zone; YR, the Yellow River zone; and NE, the Northeast zone; ^b^ Temp was the average temperature for 60 days before harvest; the temperature of everyday was obtained from the historical weather of weather network [42].

## References

[1] Statistical Yearbook of China. http://www.stats.gov.cn/tjsj/ndsj/2016/indexch.htm/.

[2] FAOSTAT: Crop. http://www.fao.org/faostat/zh/#data/QC.

[3] Yu S.C.W. (2008). Peanut varieties and their genealogy in china.

[4] Barros G., Torres A., Chulze S. (2005). Aspergillus flavus population isolated from soil of Argentina's peanut-growing region. Sclerotia production and toxigenic profile. J. Sci. Food Agric..

[5] Dorner J.W. (2008). Managent and prevention of mycotoxins in peanuts. Food Addit. Contam.-Part A Chem. Anal. Control Exp. Risk Assess..

[6] Torres A.M., Rodriguez M.I., Chulze S.N. (2006). Genetic diversity within aspergillus flavus strains isolated from peanut-cropped soils in argentina. Soil Biol. Biochem..

[7] Mahgubi A.E., Puel O., Bailly S., Tadrist S., Querin A., Ouadia A., Oswald I.P., Bailly J.D. (2013). Distribution and toxigenicity of aspergillus section flavi in spices marketed in Morocco. Food Control.

[8] Fountain J.C., Scully B.T., Chen Z.Y., Gold S.E., Glenn A.E., Abbas H.K., Lee R.D., Kemerait R.C., Guo B. (2015). Effects of hydrogen peroxide on different toxigenic and atoxigenic isolates of aspergillus flavus. Toxins.

[9] Ortega-Beltran A., Jaime R., Cotty P.J. (2015). Aflatoxin-producing fungi in maize field soils from sea level to over 2000masl: A three year study in sonora, mexico. Fungal Biol..

[10] Kana J.R., Harvey J., Wainaina J., Wanjuki I., Skilton R.A., Teguia A. (2013). Assessment of aflatoxin contamination of maize, peanut meal and poultry feed mixtures from different agroecological zones in cameroon. Toxins.

[11] Ding X., Li P., Bai Y., Zhou H. (2012). Aflatoxin b 1 in post-harvest peanuts and dietary risk in china. Food Control.

[12] Cotty P.J. (1997). Aflatoxin-producing potential of communities of aspergillus section flavi from cotton producing areas in the united states. Mycol. Res..

[13] Horn B.W., Dorner J.W. (1999). Regional differences in production of aflatoxin b1 and cyclopiazonic acid by soil isolates of aspergillus flavus along a transect within the united states. Appl. Environ. Microbiol..

[14] Williams J.H., Phillips T.D., Jolly P.E., Stiles J.K., Jolly C.M., Aggarwal D. (2004). Human aflatoxicosis in developing countries: A review of toxicology, exposure, potential health consequences, and interventions. Am. J. Clin. Nutr..

[15] Tran-Dinh N., Kennedy I., Bui T., Carter D. (2009). Survey of vietnamese peanuts, corn and soil for the presence of aspergillus flavus and aspergillus parasiticus. Mycopathologia.

[16] Giorni P., Magan N., Pietri A., Bertuzzi T., Battilani P. (2007). Studies on aspergillus section flavi isolated from maize in northern italy. Int. J. Food Microbiol..

[17] Sweany R.R., Damann K.E., Kaller M.D. (2011). Comparison of soil and corn kernel aspergillus flavus populations: Evidence for niche specialization. Phytopathology.

[18] Amani S., Shams-Ghahfarokhi M., Banasaz M., Razzaghi-Abyaneh M. (2012). Mycotoxin-producing ability and chemotype diversity of aspergillus section flavi from soils of peanut-growing regions in iran. Indian J. Microbiol..

[19] Atayde D.D., Reis T.A., Godoy I.J., Zorzete P., Reis G.M., Corrêa B. (2012). Mycobiota and aflatoxins in a peanut variety grown in different regions in the state of são paulo, brazil. Crop Protect..

[20] Nesci A., Montemarani A., Etcheverry M. (2011). Assessment of mycoflora and infestation of insects, vector of aspergillus section flavi, in stored peanut from argentina. Mycotoxin Res..

[21] Jaimegarcia R., Cotty P.J. (2007). Aspergillus flavus in soils and corncobs in south texas: Implications for management of aflatoxins in corn-cotton rotations. Plant Dis..

[22] Scarpari M., Bello C., Pietricola C., Zaccaria M., Bertocchi L., Angelucci A., Ricciardi M.R., Scala V., Parroni A., Fabbri A.A. (2014). Aflatoxin control in maize by trametes versicolor. Toxins.

[23] Astoreca A., Vaamonde G., Dalcero A., Marin S., Ramos A. (2014). Abiotic factors and their interactions influence on the co-production of aflatoxin b 1 and cyclopiazonic acid by aspergillus flavus isolated from corn. Food Microbiol..

[24] Joffe A.Z. (1969). Aflatoxin produced by 1,626 isolates of aspergillus flavus from groundnut kernels and soils in israel. Nature.

[25] Pinto V.F., Patriarca A., Locani O., Vaamonde G. (2001). Natural co-occurrence of aflatoxin and cyclopiazonic acid in peanuts grown in argentina. Food Addit. Contam..

[26] Ehrlich K.C., Kobbeman K., Montalbano B.G., Cotty P.J. (2007). Aflatoxin-producing aspergillus species from thailand. Int. J. Food Microbiol..

[27] Donner M., Atehnkeng J., Sikora R.A., Bandyopadhyay R., Cotty P.J. (2009). Distribution of aspergillus section flavi in soils of maize fields in three agroecological zones of nigeria. Soil Biol. Biochem..

[28] Shearer J.F., Sweets L.E., Baker N.K., Tiffany L.H. (1992). A study of aspergillus flavus/parasiticus in iowa crop fields: 1988–1990. Plant Dis..

[29] Zorzete P., Reis T.A., Felício J.D., Baquião A.C., Makimoto P., Corrêa B. (2011). Fungi, mycotoxins and phytoalexin in peanut varieties, during plant growth in the field. Food Chem..

[30] Orum T.V., Bigelow D.M., Nelson M.R., Howell D.R., Cotty P.J. (2007). Spatial and temporal patterns of aspergillus flavus strain composition and propagule density in yuma county, arizona, soils. Plant Dis..

[31] Antilla L., Cotty P. (2002). The ars-acrpc partnership to control aflatoxin in arizona cotton: Current status. Mycopathologia.

[32] Nesci A., Etcheverry M. (2002). Aspergillus section flavi populations from field maize in argentina. Lett. Appl. Microbiol..

[33] Horn B., Dorner J. (1998). Soil populations of aspergillus species from section flavi along a transect through peanut-growing regions of the united states. Mycologia.

[34] Garber R.K., Cotty P.J. (1997). Formation of sclerotia and aflatoxins in developing cotton bolls infected by the s strain of aspergillus flavus and potential for biocontrol with an atoxigenic strain. Phytopathology.

[35] Pildain M.B., Vaamonde G., Cabral D. (2004). Analysis of population structure of aspergillus flavus from peanut based on vegetative compatibility, geographic origin, mycotoxin and sclerotia production. Int. J. Food Microbiol..

[36] Razzaghi-Abyaneh M., Shams-Ghahfarokhi M., Allameh A., Kazeroon-Shiri A., Ranjbar-Bahadori S., Mirzahoseini H., Rezaee M.B. (2006). A survey on distribution of aspergillus section flavi in corn field soils in iran: Population patterns based on aflatoxins, cyclopiazonic acid and sclerotia production. Mycopathologia.

[37] Novas M.V., Cabral D. (2002). Association of mycotoxin and sclerotia production with compatibility groups in aspergillus flavus from peanut in argentina. Plant Dis..

[38] Okoth S., Nyongesa B., Ayugi V., Kang’Ethe E., Korhonen H., Joutsjoki V. (2012). Toxigenic potential of aspergillus species occurring on maize kernels from two agro-ecological zones in kenya. Toxins.

[39] Ding X., Wu L., Li P., Zhang Z., Zhou H., Bai Y., Chen X., Jiang J. (2014). Risk assessment on dietary exposure to aflatoxin b₁ in post-harvest peanuts in the yangtze river ecological region. Toxins.

[40] Mylroie J.E., Ozkan S., Shivaji R., Windham G.L., Alpe M.N., Williams W.P. (2016). Identification and quantification of a toxigenic and non-toxigenic aspergillus flavus strain in contaminated maize using quantitative real-time pcr. Toxins.

[41] Yin Y., Lou T., Yan L., Michailides T.J., Ma Z. (2009). Molecular characterization of toxigenic and atoxigenic aspergillus flavus isolates, collected from peanut fields in china. J. Appl. Microbiol..

[42] Weather History. http://lishi.tianqi.com/.

[43] Cotty D.P.J. (1994). Comparison of four media for the isolation of aspergillus flavus group fungi. Mycopathologia.

[44] Rodrigues P., Santos C., Venâncio A., Lima N. (2011). Species identification of aspergillus section flavi isolates from portuguese almonds using phenotypic, including maldi-tof icms, and molecular approaches. J. Appl. Microbiol..

[45] Horn B.W., Layton R.C. (1996). Association of morphology and mycotoxin production with vegetative compatibility groups in aspergillus flavus, a. Parasiticus, and a. Tamarii. Mycologia.

